# Penta­cyclo­[8.2.1.1^4,7^.0^2,9^.0^3,8^]tetra­deca-5,11-diene

**DOI:** 10.1107/S1600536812038780

**Published:** 2012-09-19

**Authors:** Hsing-Yang Tsai, Ming-Hui Luo, Wei-Chi Lin, Che-Wei Chang, Kew-Yu Chen

**Affiliations:** a Department of Chemical Engineering, Feng Chia University, 40724 Taichung, Taiwan

## Abstract

The title compound, C_14_H_16_, was prepared through [2 + 2] cyclo­addition of norbornadiene. There are two independent mol­ecules in the asymmetric unit: each is centrosymmetric with the centroid of the four-membered ring located about an inversion center. Each mol­ecule possesses an *exo–trans–exo* conformation.

## Related literature

For the preparation of the title compound, see: Chen *et al.* (2002 ▶). For the spectroscopy of D–S–A mol­ecules (electron donor–acceptor chromophores linked by spacers), see: Chen *et al.* (2002 ▶, 2006 ▶); Chow *et al.* (1999 ▶, 2005 ▶). For the electronic device applications of D–S–A mol­ecules, see: Huang *et al.* (2011 ▶); Lee *et al.* (2011 ▶); Lin *et al.* (2010 ▶); Raposo *et al.* (2011 ▶); Wang *et al.* (2011 ▶); Wu *et al.* (2010 ▶); Xiang *et al.* (2011 ▶); Zhou *et al.* (2011 ▶). For related structures, see: Chen *et al.* (2011*a* ▶,*b* ▶); Tsai *et al.* (2012 ▶). For puckering parameters, see: Cremer & Pople (1975 ▶).
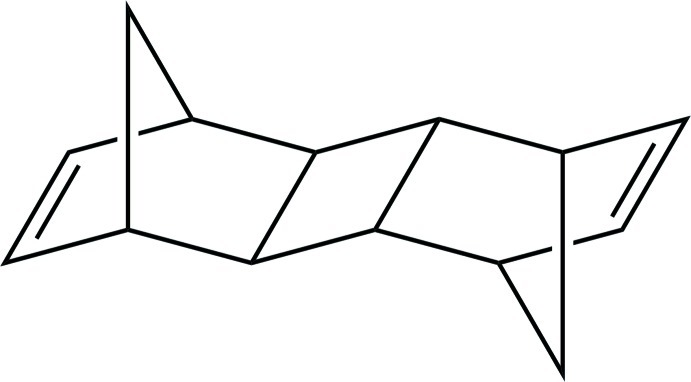


## Experimental

### 

#### Crystal data

C_14_H_16_
*M* *_r_* = 184.27
Monoclinic, 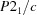

*a* = 10.7893 (7) Å
*b* = 10.8730 (6) Å
*c* = 9.2407 (6) Å
β = 109.022 (7)°
*V* = 1024.85 (11) Å^3^
*Z* = 4
Mo *K*α radiation
μ = 0.07 mm^−1^
*T* = 297 K
0.70 × 0.60 × 0.50 mm

#### Data collection

Bruker SMART CCD area-detector diffractometer
4696 measured reflections
2375 independent reflections
1662 reflections with *I* > 2σ(*I*)
*R*_int_ = 0.014

#### Refinement

*R*[*F* ^2^ > 2σ(*F* ^2^)] = 0.053
*wR*(*F* ^2^) = 0.157
*S* = 1.07
2375 reflections
127 parameters
H-atom parameters constrained
Δρ_max_ = 0.26 e Å^−3^
Δρ_min_ = −0.20 e Å^−3^

### 

Data collection: *SMART* (Bruker, 2001 ▶); cell refinement: *SAINT* (Bruker, 2001 ▶); data reduction: *SAINT*; program(s) used to solve structure: *SHELXS97* (Sheldrick, 2008 ▶); program(s) used to refine structure: *SHELXL97* (Sheldrick, 2008 ▶); molecular graphics: *ORTEP-3 for Windows* (Farrugia, 1997 ▶); software used to prepare material for publication: *WinGX* (Farrugia, 1999 ▶).

## Supplementary Material

Crystal structure: contains datablock(s) I, global. DOI: 10.1107/S1600536812038780/xu5620sup1.cif

Structure factors: contains datablock(s) I. DOI: 10.1107/S1600536812038780/xu5620Isup2.hkl

Supplementary material file. DOI: 10.1107/S1600536812038780/xu5620Isup3.cml

Additional supplementary materials:  crystallographic information; 3D view; checkCIF report

## Figures and Tables

**Fig. 1.**
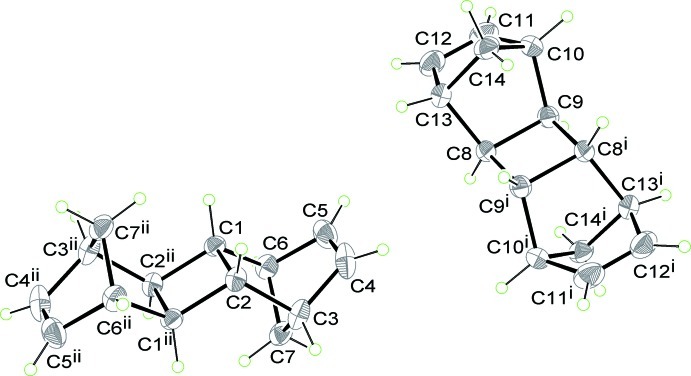
The molecular structure of the title compound, showing 50% probability displacement ellipsoids.

## supplementary crystallographic information

### Comment

Electron donor (D)-acceptor (A) chromophores linked by spacers (S), forming D–S–A dyads (Huang *et al.*, 2011; Lee *et al.*, 2011; Raposo *et al.*, 2011), have attracted considerable attention due to their potential applications in the design of molecular devices (Lin *et al.*, 2010; Wang *et al.*, 2011; Wu *et al.*, 2010; Xiang *et al.*, 2011; Zhou *et al.*, 2011). Numerous types of rigid spacers have also been reported (Chen *et al.*, 2002; Chow *et al.*, 1999). The highly symmetrical structures reduce the complexity due to the constraint of geometrical and conformational variations. Consequently, the rates of photoinduced electron transfer reactions across linearly fused oligo-norbornyl spacer groups can be extensively investigated (Chen *et al.*, 2006; Chow *et al.*, 2005).

The *ORTEP* diagram of the title compound is shown in Figure 1. There are two crystallographically independent molecules in the asymmetric unit. The molecules possess an *exo-trans-exo* configuration. The puckering parameters (Cremer & Pople, 1975) of the five-membered rings *A* (C1–C3/C7/C6) and *B* (C3–C7) are *Q*_2_ = 0.5975 (16) Å and φ_2_ = 287.85 (15)°, and *Q*_2_ = 0.5504 (17) Å and φ_2_ = 144.42 (18)°, respectively. These results are slightly different from those of previous studies on other norbornane derivatives (Chen, *et al.*, 2011*a*,*b*, 2002).

### Experimental

The title compound was synthesized according to the literature (Chen *et al.*, 2002). Colorless parallelepiped-shaped crystals suitable for the crystallographic studies reported here were isolated over a period of five weeks by slow evaporation from a chloroform solution.

### Refinement

The C bound H atoms positioned geometrically and allowed to ride on their parent atoms, with *U*_iso_(H) = 1.2*U*_eq_(C).

### Crystal data

**Table:**

C_14_H_16_	*F*(000) = 400
*M**_r_* = 184.27	*D*_x_ = 1.194 Mg m^−^^3^
Monoclinic, *P*2_1_/*c*	Mo *K*α radiation, λ = 0.71073 Å
Hall symbol: -P 2ybc	Cell parameters from 2538 reflections
*a* = 10.7893 (7) Å	θ = 3.0–29.2°
*b* = 10.8730 (6) Å	µ = 0.07 mm^−^^1^
*c* = 9.2407 (6) Å	*T* = 297 K
β = 109.022 (7)°	Parallelepiped, colorless
*V* = 1024.85 (11) Å^3^	0.70 × 0.60 × 0.50 mm
*Z* = 4	

### Data collection

**Table:**

Bruker SMART CCD area-detector diffractometer	1662 reflections with *I* > 2σ(*I*)
Radiation source: fine-focus sealed tube	*R*_int_ = 0.014
Graphite monochromator	θ_max_ = 29.2°, θ_min_ = 3.0°
ω scans	*h* = −13→14
4696 measured reflections	*k* = −13→14
2375 independent reflections	*l* = −12→10

### Refinement

**Table:**

Refinement on *F*^2^	Primary atom site location: structure-invariant direct methods
Least-squares matrix: full	Secondary atom site location: difference Fourier map
*R*[*F*^2^ > 2σ(*F*^2^)] = 0.053	Hydrogen site location: inferred from neighbouring sites
*wR*(*F*^2^) = 0.157	H-atom parameters constrained
*S* = 1.07	*w* = 1/[σ^2^(*F*_o_^2^) + (0.098*P*)^2^ + 0.0079*P*] where *P* = (*F*_o_^2^ + 2*F*_c_^2^)/3
2375 reflections	(Δ/σ)_max_ = 0.001
127 parameters	Δρ_max_ = 0.26 e Å^−^^3^
0 restraints	Δρ_min_ = −0.20 e Å^−^^3^

### Special details

**Table:**

Geometry. All e.s.d.'s (except the e.s.d. in the dihedral angle between two l.s. planes) are estimated using the full covariance matrix. The cell e.s.d.'s are taken into account individually in the estimation of e.s.d.'s in distances, angles and torsion angles; correlations between e.s.d.'s in cell parameters are only used when they are defined by crystal symmetry. An approximate (isotropic) treatment of cell e.s.d.'s is used for estimating e.s.d.'s involving l.s. planes.
Refinement. Refinement of *F*^2^ against ALL reflections. The weighted *R*-factor *wR* and goodness of fit *S* are based on *F*^2^, conventional *R*-factors *R* are based on *F*, with *F* set to zero for negative *F*^2^. The threshold expression of *F*^2^ > σ(*F*^2^) is used only for calculating *R*-factors(gt) *etc*. and is not relevant to the choice of reflections for refinement. *R*-factors based on *F*^2^ are statistically about twice as large as those based on *F*, and *R*- factors based on ALL data will be even larger.

### Fractional atomic coordinates and isotropic or equivalent isotropic displacement parameters (Å^2^)

**Table:**

	*x*	*y*	*z*	*U*_iso_*/*U*_eq_	
C1	0.48726 (13)	0.60019 (11)	−0.00625 (14)	0.0354 (3)	
H1A	0.4862	0.6542	0.0783	0.042*	
C2	0.60674 (12)	0.51050 (12)	0.03238 (15)	0.0363 (3)	
H2A	0.6678	0.5178	0.1370	0.044*	
C3	0.66729 (14)	0.54040 (14)	−0.09413 (18)	0.0493 (4)	
H3A	0.7283	0.4794	−0.1102	0.059*	
C4	0.71928 (16)	0.66991 (16)	−0.05935 (19)	0.0626 (5)	
H4A	0.8071	0.6926	−0.0217	0.075*	
C5	0.61875 (17)	0.74451 (15)	−0.09167 (18)	0.0576 (5)	
H5A	0.6226	0.8296	−0.0805	0.069*	
C6	0.49605 (14)	0.66859 (12)	−0.15005 (16)	0.0414 (4)	
H6A	0.4169	0.7122	−0.2113	0.050*	
C7	0.54463 (14)	0.56681 (14)	−0.23163 (15)	0.0455 (4)	
H7A	0.4851	0.4973	−0.2591	0.055*	
H7B	0.5651	0.5961	−0.3204	0.055*	
C8	0.89681 (12)	0.98409 (12)	−0.01155 (15)	0.0366 (3)	
H8A	0.8246	0.9749	−0.1082	0.044*	
C9	0.98405 (13)	1.09971 (11)	−0.00363 (15)	0.0377 (3)	
H9A	0.9572	1.1510	−0.0959	0.045*	
C10	0.97907 (14)	1.16558 (13)	0.14375 (18)	0.0485 (4)	
H10A	1.0466	1.2278	0.1875	0.058*	
C11	0.83794 (17)	1.20757 (15)	0.1034 (2)	0.0606 (5)	
H11A	0.8086	1.2884	0.0865	0.073*	
C12	0.76413 (15)	1.11028 (15)	0.09629 (19)	0.0572 (5)	
H12A	0.6736	1.1098	0.0736	0.069*	
C13	0.85276 (13)	0.99966 (13)	0.13162 (17)	0.0436 (4)	
H13A	0.8180	0.9259	0.1658	0.052*	
C14	0.97517 (14)	1.05643 (14)	0.24666 (16)	0.0477 (4)	
H14A	1.0517	1.0039	0.2680	0.057*	
H14B	0.9617	1.0811	0.3413	0.057*	

### Atomic displacement parameters (Å^2^)

**Table:**

	*U*^11^	*U*^22^	*U*^33^	*U*^12^	*U*^13^	*U*^23^
C1	0.0424 (7)	0.0336 (7)	0.0330 (7)	0.0020 (5)	0.0163 (6)	0.0007 (5)
C2	0.0315 (7)	0.0444 (8)	0.0324 (6)	0.0012 (5)	0.0095 (5)	0.0066 (5)
C3	0.0415 (8)	0.0599 (9)	0.0551 (9)	0.0096 (7)	0.0276 (7)	0.0171 (8)
C4	0.0453 (9)	0.0788 (12)	0.0611 (10)	−0.0188 (8)	0.0140 (8)	0.0218 (9)
C5	0.0699 (12)	0.0488 (9)	0.0535 (9)	−0.0171 (8)	0.0190 (8)	0.0093 (7)
C6	0.0450 (8)	0.0404 (7)	0.0397 (7)	0.0059 (6)	0.0151 (6)	0.0111 (6)
C7	0.0552 (9)	0.0520 (8)	0.0348 (7)	0.0004 (7)	0.0223 (7)	0.0053 (6)
C8	0.0320 (7)	0.0425 (7)	0.0348 (7)	−0.0061 (5)	0.0103 (5)	−0.0051 (5)
C9	0.0418 (8)	0.0336 (7)	0.0389 (7)	−0.0026 (5)	0.0150 (6)	0.0015 (5)
C10	0.0528 (9)	0.0404 (8)	0.0573 (9)	−0.0115 (6)	0.0249 (7)	−0.0150 (7)
C11	0.0694 (12)	0.0495 (9)	0.0710 (11)	0.0158 (8)	0.0340 (9)	−0.0025 (8)
C12	0.0446 (9)	0.0711 (12)	0.0600 (10)	0.0089 (8)	0.0228 (8)	−0.0068 (8)
C13	0.0410 (8)	0.0490 (8)	0.0464 (8)	−0.0058 (6)	0.0217 (7)	−0.0023 (6)
C14	0.0481 (9)	0.0603 (9)	0.0363 (7)	−0.0013 (7)	0.0159 (6)	−0.0069 (7)

### Geometric parameters (Å, º)

**Table:**

C1—C6	1.5525 (16)	C8—C9^ii^	1.5443 (17)
C1—C2^i^	1.5413 (17)	C8—C13	1.5539 (18)
C1—C2	1.5621 (17)	C8—C9	1.5578 (17)
C1—H1A	0.9800	C8—H8A	0.9800
C2—C1^i^	1.5413 (17)	C9—C10	1.5551 (18)
C2—C3	1.5483 (17)	C9—C8^ii^	1.5442 (17)
C2—H2A	0.9800	C9—H9A	0.9800
C3—C4	1.511 (2)	C10—C11	1.515 (2)
C3—C7	1.534 (2)	C10—C14	1.530 (2)
C3—H3A	0.9800	C10—H10A	0.9800
C4—C5	1.309 (2)	C11—C12	1.313 (2)
C4—H4A	0.9300	C11—H11A	0.9300
C5—C6	1.503 (2)	C12—C13	1.505 (2)
C5—H5A	0.9300	C12—H12A	0.9300
C6—C7	1.5249 (19)	C13—C14	1.5298 (19)
C6—H6A	0.9800	C13—H13A	0.9800
C7—H7A	0.9700	C14—H14A	0.9700
C7—H7B	0.9700	C14—H14B	0.9700
			
C6—C1—C2^i^	117.43 (11)	C9^ii^—C8—C13	117.61 (11)
C6—C1—C2	102.62 (9)	C9^ii^—C8—C9	89.98 (9)
C2^i^—C1—C2	90.01 (9)	C13—C8—C9	102.70 (10)
C6—C1—H1A	114.5	C9^ii^—C8—H8A	114.4
C2^i^—C1—H1A	114.5	C13—C8—H8A	114.4
C2—C1—H1A	114.5	C9—C8—H8A	114.4
C1^i^—C2—C3	117.63 (11)	C10—C9—C8^ii^	117.23 (12)
C1^i^—C2—C1	89.99 (9)	C10—C9—C8	102.70 (9)
C3—C2—C1	102.49 (10)	C8^ii^—C9—C8	90.02 (9)
C1^i^—C2—H2A	114.5	C10—C9—H9A	114.6
C3—C2—H2A	114.5	C8^ii^—C9—H9A	114.6
C1—C2—H2A	114.5	C8—C9—H9A	114.6
C4—C3—C7	99.32 (12)	C11—C10—C9	104.01 (12)
C4—C3—C2	104.70 (12)	C11—C10—C14	99.07 (12)
C7—C3—C2	101.67 (10)	C9—C10—C14	101.71 (10)
C4—C3—H3A	116.2	C11—C10—H10A	116.5
C7—C3—H3A	116.2	C9—C10—H10A	116.5
C2—C3—H3A	116.2	C14—C10—H10A	116.5
C5—C4—C3	107.85 (14)	C12—C11—C10	108.30 (14)
C5—C4—H4A	126.1	C12—C11—H11A	125.8
C3—C4—H4A	126.1	C10—C11—H11A	125.8
C4—C5—C6	108.00 (14)	C11—C12—C13	107.52 (13)
C4—C5—H5A	126.0	C11—C12—H12A	126.2
C6—C5—H5A	126.0	C13—C12—H12A	126.2
C5—C6—C7	99.87 (11)	C12—C13—C14	99.90 (12)
C5—C6—C1	104.38 (11)	C12—C13—C8	104.56 (11)
C7—C6—C1	101.67 (10)	C14—C13—C8	101.61 (9)
C5—C6—H6A	116.2	C12—C13—H13A	116.1
C7—C6—H6A	116.2	C14—C13—H13A	116.1
C1—C6—H6A	116.2	C8—C13—H13A	116.1
C3—C7—C6	93.97 (11)	C13—C14—C10	94.21 (11)
C3—C7—H7A	112.9	C13—C14—H14A	112.9
C6—C7—H7A	112.9	C10—C14—H14A	112.9
C3—C7—H7B	112.9	C13—C14—H14B	112.9
C6—C7—H7B	112.9	C10—C14—H14B	112.9
H7A—C7—H7B	110.3	H14A—C14—H14B	110.3
			
C6—C1—C2—C1^i^	−118.17 (11)	C9^ii^—C8—C9—C10	−117.97 (12)
C2^i^—C1—C2—C1^i^	0.0	C13—C8—C9—C10	0.39 (13)
C6—C1—C2—C3	0.19 (13)	C9^ii^—C8—C9—C8^ii^	0.0
C2^i^—C1—C2—C3	118.36 (12)	C13—C8—C9—C8^ii^	118.36 (12)
C1^i^—C2—C3—C4	163.80 (12)	C8^ii^—C9—C10—C11	−164.01 (12)
C1—C2—C3—C4	67.16 (13)	C8—C9—C10—C11	−67.32 (13)
C1^i^—C2—C3—C7	60.79 (15)	C8^ii^—C9—C10—C14	−61.43 (13)
C1—C2—C3—C7	−35.86 (14)	C8—C9—C10—C14	35.25 (13)
C7—C3—C4—C5	33.64 (15)	C9—C10—C11—C12	71.17 (16)
C2—C3—C4—C5	−71.14 (15)	C14—C10—C11—C12	−33.41 (16)
C3—C4—C5—C6	−0.36 (17)	C10—C11—C12—C13	0.03 (18)
C4—C5—C6—C7	−33.32 (15)	C11—C12—C13—C14	33.45 (15)
C4—C5—C6—C1	71.54 (14)	C11—C12—C13—C8	−71.41 (15)
C2^i^—C1—C6—C5	−164.42 (11)	C9^ii^—C8—C13—C12	164.42 (11)
C2—C1—C6—C5	−67.74 (12)	C9—C8—C13—C12	67.68 (13)
C2^i^—C1—C6—C7	−60.91 (14)	C9^ii^—C8—C13—C14	60.85 (14)
C2—C1—C6—C7	35.78 (12)	C9—C8—C13—C14	−35.90 (13)
C4—C3—C7—C6	−50.28 (11)	C12—C13—C14—C10	−50.63 (11)
C2—C3—C7—C6	56.97 (12)	C8—C13—C14—C10	56.62 (12)
C5—C6—C7—C3	50.32 (11)	C11—C10—C14—C13	50.10 (12)
C1—C6—C7—C3	−56.74 (11)	C9—C10—C14—C13	−56.37 (11)

Symmetry codes: (i) −*x*+1, −*y*+1, −*z*; (ii) −*x*+2, −*y*+2, −*z*.
